# Regression of Post-Essential Thrombocythaemia Myelofibrosis with Intermittent Hydroxyurea Therapy: A Case Report

**DOI:** 10.3390/jcm14248641

**Published:** 2025-12-05

**Authors:** Arumugam Manoharan, Ian Tang

**Affiliations:** 1 Southern Sydney Haematology, Faculty of Science, Medicine and Health, University of Wollongong, Wollongong, NSW 2500, Australia; 2 Douglas Hanley Moir Pathology, Sydney, NSW 2093, Australia; itang@dhm.com.au

**Keywords:** essential thrombocythaemia, myelofibrosis, intermittent hydroxyurea therapy, fibrosis regression

## Abstract

We describe a patient with post-essential thrombocythaemia myelofibrosis treated with intermittent hydroxyurea (Hu) therapy (20 mg/Kg, given as a single dose, thrice weekly), achieving sustained disease control and regression of bone marrow fibrosis. Additionally, we discuss the efficacy of and rationale for use of intermittent Hu therapy in patients with myeloproliferative neoplasms, including those deemed to be Hu-resistant or intolerant to the commonly used continuous therapy.

## 1. Introduction

Essential thrombocythaemia (ET) is one of four chronic myeloproliferative neoplasms (MPN), characterised by clonal thrombocytosis [1]; approximately 85% of patients will have a pathognomonic driver mutation at presentation—JAK-2 (about 50%), CALR (about 30%) or MPL (about 5%) [2]. Hydroxyurea (Hu), given as continuous therapy at a starting dose of 500 mg once or twice daily, is a commonly used first-line cytoreductive treatment for patients with ET [3]. CALR-positive ET patients generally have a good prognosis, the median survival being 20 years; patients less than 65 years old have a better median survival at 32 years [4]. However, these patients are at a higher risk of progression to myelofibrosis than those with the JAK-2 mutation [5,6,7], especially if they are resistant or intolerant to Hu therapy [8]. The resulting clinical entity, referred to as post-ET myelofibrosis [9], has an adverse impact on patient survival—even “low risk” patients (according to the Dynamic International Prognostic Scoring System plus -DIPSS+: age < 65 yrs, WBC < 25 × 10^9^/L, Hb > 100 g/L, blasts < 1%, no constitutional symptoms and normal karyotype) [10]—have a median survival of only 15 years [4]. Current therapeutic guidelines for post-ET myelofibrosis patients comprise initiation of a JAK-2 inhibitor (e.g., ruxolitinib) and consideration of stem cell transplant as definitive treatment [11,12]. In this report, we describe a patient with CALR-positive post-ET myelofibrosis treated with intermittent Hu (20 mg/Kg, given as a single dose, thrice weekly) [13], achieving effective disease control, including regression of bone marrow fibrosis from grade 3 to grade 1 [14].

## 2. Case Report

**Figure 1:**
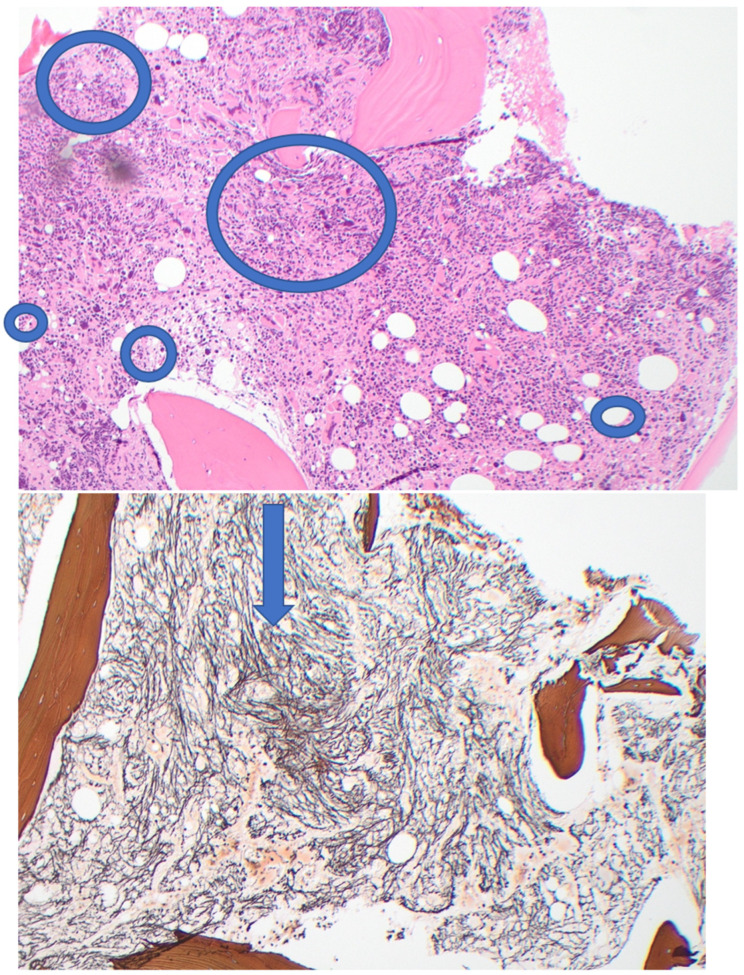
Pre-treatment bone marrow biopsy. (H&E ×10) showing marked hyper-cellularity and numerous abnormal megakaryocytes; (reticulin stain ×10) showing dense (Grade 3) fibrosis.

In December 2022, a 56 year-old female presented to our Centre for management of post-ET myelofibrosis. Five years earlier, she had been diagnosed with type 2 CALR-positive ET at another centre: Hb 112 g/L, WBC 6.3 × 10^9^/L, and platelets 1200 × 10^9^/L. She had been treated with HU 500 mg daily but this was discontinued after four months because of lack of response and neutropenia. She remained under regular reviews without any further treatment. In November 2022, she was found to be anaemic; Hb 104 g/L, WBC 9.1 × 10^9^/L, and platelets 921 × 10^9^/L. Bone marrow examination showed marked hyper-cellularity, reduced erythropoiesis, hypo-granular neutrophils, dysplastic megakaryocytes, and markedly increased fibrosis (grade 3) [14]—see Figure 1. She was assessed to be in the “low risk” prognostic category and “not-a-candidate” for allogenic stem cell transplantation because of potential procedure-related morbidity.

At presentation to our Centre, she was clinically well but anxious about the possibility of thrombotic events and the progression of thrombocythaemia to myelofibrosis. Her past history included mild hypertension (treated with amlodipine) and Meniere’s disease. Physical examination was unremarkable; in particular, there was no splenomegaly.

**Figure 2:**
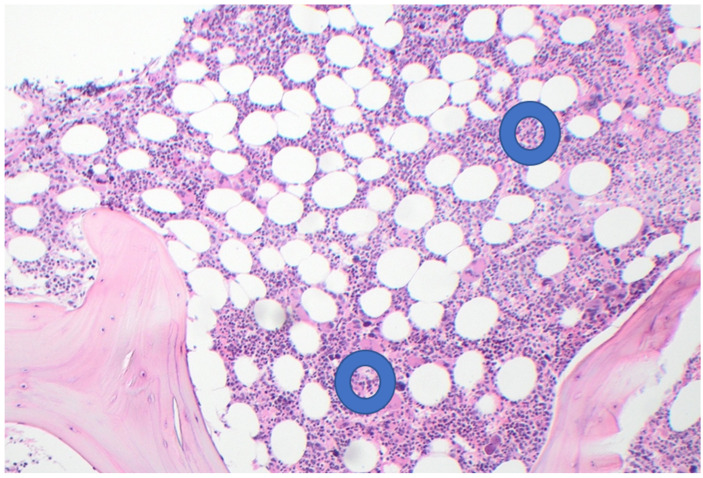

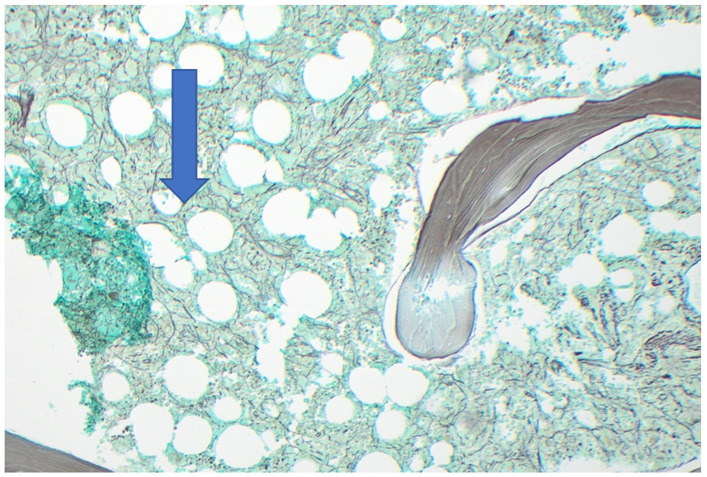
Post-treatment bone marrow biopsy. (H&E ×10) showing mild hyper-cellularity and fewer abnormal megakaryocytes; (reticulin stain ×10) showing mild (Grade 1) fibrosis.

Serum chemistry, liver function tests, uric acid, TSH, and iron studies were all normal. Whole blood platelet aggregation studies showed features of platelet hyperactivity; repeat tests after four weeks of aspirin 100 mg daily dose showed an optimum anti-platelet effect [15]. She was considered to be a candidate for concomitant cytoreductive therapy to control the disease activity (i.e., thrombocytosis) and to slow down its progression. She was commenced on hydroxyurea 1.5 g (20 g/Kg) given as a single dose, thrice weekly. She tolerated the treatment well and remained under 2–3 monthly reviews. Twelve months later, blood counts showed Hb 128 g/L, WBC 2.7 × 10^9^/L (Neutrophils 1.1), and platelets 662 × 10^9^/L. These numbers remained stable over the next 18 months. A repeat bone marrow examination in May 2025 showed mild hyper-cellularity, adequate erythropoiesis, and normal myelopoiesis with occasional hypo-granular neutrophils and increased number of megakaryocytes with hyper-/hypo-lobulated nuclei; reticulin stain showed grade 1 of 3 fibrosis [14]—see Figure 2. Cytogenetic studies were normal. Gene panel studies showed variants in TET 2, CALR (type 2), and DNMT3 A, consistent with myeloproliferative neoplasm (MPN). At the time of writing (October 2025), she remains on Hu and aspirin therapies and leads an active life, with a Karnofsky performance status score of 100% [16]. Her current blood counts and all the pre- and post-treatment haematological parameters are summarised in Table 1.

## 3. Discussion

The aim of treatment in patients with ET is to prevent thrombosis and to slow down or delay disease progression. Our patient’s concern for thrombotic events is common among ET patients. A survey by Mesa et al. in the United States found thrombosis prevention to be the most important goal of treatment for 35% of patients with ET and 57% of physicians treating ET patients [17]. At our Centre, we routinely use whole blood platelet aggregation studies in all MPN patients for risk assessment and to tailor anti-platelet therapy in individual patients. In a study of 132 patients (including 98 ET patients), we have documented varying degrees of platelet hyperactivity in 115 patients (87 ET patients) and a thrombosis-free survival rate of 1–23 years (median 8 years) whilst on the individualised anti-platelet therapy [15]; no significant differences were noted in terms of aspirin dose requirements (ranging from 100 mg twice or thrice weekly to 400 mg daily) between JAK-2, CALR- or MPL-positive patients and among the four IPSET-thrombosis [18,19] sub-groups.

Hu is a commonly used first-line therapeutic agent for cytoreduction in patients with MPN [20,21,22], the most used schedule being 500 mg twice daily and titrated on the basis of response and blood counts [3,23,24]. However, several studies have reported resistance, intolerance or disease progression in 10–30% of patients receiving Hu as continuous therapy [25,26]. A recent Spanish study of 1080 ET patients has reported inferior clinical outcomes in those with resistance or intolerance to first-line Hu therapy [8].

At our Centre, we have used Hu as intermittent therapy (akin to schedules used in patients with solid tumours) at 20–30 mg/kg doses, given as a single dose, twice or thrice weekly. During the past 30 years, we have treated 118 MPN patients (polycythaemia vera-29, ET-84, primary myelofibrosis-5) and have observed (median follow-up 8.5 years) sustained responses without troublesome cytopenias or the need for treatment interruptions [27]. The total weekly dose of intermittent Hu used at our Centre is comparable to those commonly recommended in the continuous regimen. Based on the review of pharmaco-kinetics [28,29] of Hu and our experience, we hypothesise that the better clinical outcome with intermittent therapy is attributable to (i) a higher plasma level of Hu achieved with the single dose intake (more than 80% of orally administered dose of Hu is readily absorbed, reaching peak plasma levels in 1–4 h); (ii) the preferential uptake of Hu by the mitotically more active clonal proliferative cells; and, (iii) the unhindered, normal haemopoietic activity on treatment-free days each week (the plasma half-life of Hu is short—2–4 h).

Recent review articles on the management of patients with post-ET myelofibrosis recommend prognostic stratification using a clinical-molecular prognostic model to predict survival [30,31], treatment with a JAK-2 inhibitor agent (ruxolitinib, fedratinib, pacritinib, momelotinib or jaktinib), and early consideration of stem cell transplant, especially for patients in the “low risk” category [11,12,32]. JAK-2 inhibitor therapy, currently considered to be the mainstay-of-treatment for myelofibrosis, can be effective in reducing splenic volume and minimise disease-related symptoms (thus improving the quality of life) even in patients with CALR-positive post-ET myelofibrosis [33]; however, these agents have no effect on the underlying clonal proliferation, nor can they change the course of the disease. The patient described in this report did not have splenomegaly or any disease-related symptoms. Her clinical picture comprised persistent thrombocytosis, anaemia, and dense bone marrow fibrosis. Intermittent Hu therapy, through its action on the proliferating clone, has been effective, achieving improvement (albeit incomplete) of all these parameters.

Bone marrow fibrosis in patients with MPN is a reactive process [34], attributable to overproduction of abnormal megakaryocytes and excessive release of cytokines that stimulate fibroblasts to deposit collagen, resulting in fibrosis [35]. Reversibility of this reactive process has been well documented with effective chemotherapy, including Hu [36,37,38,39]. Continuous Hu therapy at a starting dose of 500 mg daily, however, is commonly associated with worsening anaemia or cytopenias, necessitating change in treatment in 80% of patients at 12 months [21]. The patient described in this report has had no clinical problems with the intermittent therapy over a 2½ year period. Regression of fibrosis with effective chemotherapy suggests that the decrease in the number of megakaryocytes and also possibly the release of pathogenic cytokines enable normal collagenase activity in the marrow micro-environment to gradually reduce fibrosis.

The favourable clinical outcome with the intermittent Hu therapy in this report should be seen in the context of the following limitations: (i) this is a single patient/case study and (ii) the patient did not have any disease-related symptoms. However, intermittent Hu has been used to treat MF patients at our Centre for more than 30 years [27]. In a study published in 1991, we reported beneficial effects (resolution of constitutional symptoms, reduction in spleen size, improved haemoglobin level) in eight out of ten myelofibrosis patients treated with intermittent Hu [13]. The clinical responses observed in this study were similar/comparable to those reported by Lofvenberg et al., with continuous Hu therapy; these authors also documented reversal of bone marrow fibrosis with Hu therapy [39]. We have used JAK-2 inhibitor therapy only in patients with persistent disease-related symptoms (ruxolitinib) or anaemia (momelotinib).

The efficacy and tolerability of intermittent Hu therapy in MPN patients need to be confirmed in large, randomised studies. In the interim, we hope our publications will encourage clinicians to consider the intermittent dosage schedule (i) in the Hu-resistant or intolerant patients on continuous therapy before changing over to more invasive and/or expensive second-line therapies and (ii) in “low risk” post-ET myelofibrosis patients before embarking on stem cell transplantation as definitive therapy.

## Figures and Tables

**Table 1:** Summary of haematological parameters. Pre- and post-treatment.

Haematological Parameters	Pre-Treatment *	Post-Treatment (30 Months) **	Current ***
**Blood Counts**			
Hb (g/L)	104	121	121
WBC (10^9^ L)	9.10	3.30	2.40
Neutrophils (10^9^ L)	6.10	1.62	1.00
Lymphocytes (10^9^ L)	2.00	1.55	1.27
Platelets (10^9^ L)	921	636	540
**Bone Marrow**			
Cellularity	Markedly Hyper-cellular	Mildly Hyper-cellular	
Erythropoiesis	Reduced	Adequate	
Myelopoiesis	Increased	Adequate	
Megakaryocytes	Markedly Increased	Increased	
Fibrosis	Grade 3	Grade 1	

* 29 November 2022, ** 1 May 2025, *** 7 October 2025.

## Data Availability

The original contributions presented in this study are included in the article. Further inquiries can be directed to the corresponding author.
